# Higher-order topological corner state in a reconfigurable breathing kagome lattice consisting of magnetically coupled *LC* resonators

**DOI:** 10.1038/s41598-023-35509-6

**Published:** 2023-05-23

**Authors:** Kenichi Yatsugi, Shrinathan Esakimuthu Pandarakone, Hideo Iizuka

**Affiliations:** Toyota Central R&D Labs., Inc., Nagakute, Aichi 480-1192 Japan

**Keywords:** Engineering, Physics

## Abstract

Higher-order topological insulators are attracting attention from fundamental interest to fascinating applications, owing to the topological properties with higher-order topological corner states. Breathing kagome lattice is a prospective platform which can support higher-order topological corner states. Here, we experimentally demonstrate that higher-order topological corner states are supported in a breathing kagome lattice consisting of magnetically coupled resonant coils. The winding direction of each coil is determined to hold *C*_3_ symmetry for each triangle unit cell, enabling to emerge higher-order topological corner states. In addition, topological and trivial phases can be switched by changing the distances between the coils. The emergence of corner states in the topological phase is experimentally observed through admittance measurements. As an illustration, wireless power transfer is performed between the corner states, and between the bulk and corner states. The proposed configuration is a promising platform for not only investigating topological properties of the breathing kagome lattice but also an alternative mechanism of selective wireless power transfer.

## Introduction

Topological phases of matter have attractive properties in wave propagation and are expected to revolutionize technologies ranging from electronics^1,2^, photonics^3–7^, acoustics^8–10^, to mechanics^11–13^. According to the bulk-boundary correspondence, a conventional *d*-dimensional topological insulator supports (*d*-1)-dimensional boundary states^1,2^. On the other hand, recently discovered higher-order topological insulators (HOTIs) can support boundary states in (*d*-2)-dimensions^14–16^. For example, in the case of 2-dimensional systems, 0-dimensional corner states can appear. The corner states of HOTIs have been experimentally observed in various physical platforms^17–20^. Moreover, investigations on HOTIs are active research areas, from linear to nonlinear^21–24^, from real to synthetic dimensions^25^, and from Hermitian to non-Hermitian systems^26^.

One of the basic lattices supporting higher-order topological corner states is the breathing kagome lattice^27^. Conventionally, the topological corner states in HOTIs have been studied in a square and cubic lattice^14–16^. On the other hand, the breathing kagome lattice is based on the triangular lattice, and three corner states are observed at the three triangle corners^27^. There have been reports on various physical platforms for the breathing kagome lattice and the experimental observations of the topological corner states in the fields including photonics^28^, electromagnetics^29^, acoustics^30,31^, and electric circuits^32,33^. In those systems, topological phases can be obtained by properly tuning the inter- and intra-cell coupling in the lattice. Conventional designs of HOTIs rely on the lattice geometry. However, the flexible control of the topological phase is difficult in such fixed geometry.

On the other hand, one of the application areas of topological phases of matter that are attracting attentions is wireless power transfer. The wireless power transfer based on the analogy of topological insulators has been demonstrated in 1-dimensional systems^34–37^. The Harper and SSH chain composed of coupled *LC* resonators have been utilized for the demonstrations. The energy is localized at the edge of the one-dimensional *LC* resonator chain. The direction control and high efficiency power transfer have been demonstrated using the topological edge states. However, the wireless power transfer capability in 2-dimensional HOTI systems has not been demonstrated yet. The wireless power transfer in 2-dimensinoal configurations is prospective for applications such as charging mobile devices via walls or tables.

Here, we experimentally demonstrate that higher-order topological corner states are excited in a breathing kagome lattice consisting of magnetically coupled resonant coils. The winding direction of each coil is determined to hold *C*_3_ symmetry for the triangle unit cell, enabling to emerge higher-order topological corner states. The emergence of the topological states is experimentally observed through admittance measurements. In addition, our configuration has an advantage of reconfigurability, i.e., topological and trivial phases can be switched by changing the distances between the coils. As an illustration, wireless power transfer is performed between the corner states and between the bulk and corner state.

## Results

### Design of our breathing kagome lattice

**Figure 1:**
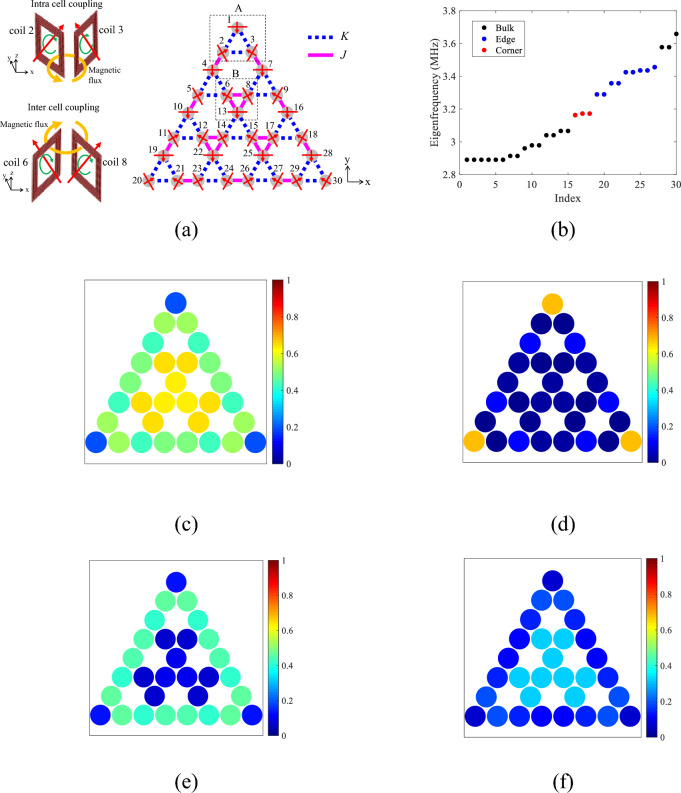
(**a**), Configuration of a breathing Kagome lattice for the arrangement of resonant coils. Each gray circle indicates the center location of a resonant coil. The top and the bottom insets show two neighboring coils for intra- and inter-cell couplings, respectively. The green arrow represents the winding direction of a coil, and the red arrow indicates the winding direction that satisfies the right-handed system. The orange arrow represents the interlinkage magnetic flux. (**b),** Calculated eigenfrequencies by using coupled mode theory. (**c**–**f)**, Calculated spatial distributions of squared values of the amplitudes of the eigenstates integrated over **c,** the 1st to the 15th (bulk), (**d),** the 16th to the 18th (corner), (**e**), the 19th to the 27th (edge), (**f**)**,** the 27th to the 30th eigen modes (bulk).

We consider a 2-dimensional arrangement of resonators, as shown in Fig. 1a. The resonators consist of identical planar spiral coils with each having the resonant frequency *ω*_0_. The gray circle indicates the center location of each coil. The red line on each circle represents the winding plane. The winding plane is perpendicular to the lattice plane (*xy* plane). The winding direction of a coil is represented by the green arrow (insets of Fig. 1a). The red arrow on each circle indicates the winding direction that satisfies the right-handed system. The blue dotted line and the pink solid line represent the intra- and inter-cell couplings labeled by *K* and *J*, respectively. Here, the resonant coils are carefully arranged considering each winding direction. In our configuration, three red arrows indicating the winding directions for each triangle unit cell of the intra-cell coupling (e.g., triangle A) direct inward to the centroid of the unit cell to hold *C*_3_ symmetry^27^. Consequently, three red arrows for each triangle of the inter-cell coupling (e.g., triangle B) direct outward from the centroid of the triangle. The sign of each coupling constant between the adjacent coils depends on the winding directions of the coupled coils^38^. For the representative intra-cell coupling (top inset of Fig. 1a), the red arrow of coil 2 (coil 3) has the opposite (same) direction as the orange arrow representing the interlinkage magnetic flux. Likewise, for the inter-cell coupling (bottom inset of Fig. 1a), the red arrow of coil 6 (coil 8) has the same (opposite) direction as the orange arrow. When magnetically-coupled two coils have opposite winding directions to the interlinkage magnetic flux, the mutual inductance has the minus sign^38^. Thus, all of the coupling constants for both intra- and inter-cell couplings have the minus sign in our configuration.

We begin with calculating eigenfrequencies of the breathing kagome lattice by using coupled mode theory (CMT) for the guide of our experimental design^39^. We assume the exp(*iωt*) convention, where *ω* and *t* are the angular frequency and time. In CMT, the dynamics of the system is described as

1a $$d\mathbf{a}/dt=\mathrm{A}\mathbf{a},$$

1b $$\mathrm{A}={\left(\begin{array}{cccccc}i{\omega }_{0}& iK& iK& 0& 0& \cdots \\ iK& i{\omega }_{0}& iK& iJ& 0& \cdots \\ iK& iK& i{\omega }_{0}& 0& 0& \cdots \\ 0& iJ& 0& i{\omega }_{0}& iK& \cdots \\ 0& 0& 0& iK& i{\omega }_{0}& \cdots \\ \vdots & \vdots & \vdots & \vdots & \vdots & \ddots \end{array}\right)}_{N\times N},$$

where $$\mathbf{a}={\left[\begin{array}{ccccc}{a}_{1}& {a}_{2}& {a}_{3}& \cdots & {a}_{N}\end{array}\right]}^{T}$$. *a*_n_ is the complex amplitude of a single mode for the *n*th resonator. Eigenfrequencies for the configuration of Fig. 1a are calculated and shown in Fig. 1b, where *ω*_0_/2π = 3.17 MHz, *K* = − 0.0615*ω*_0_, and *J* = − 0.0265*ω*_0_, and *N* = 30 are used. In a breathing kagome lattice, the topological corner states appear within the band gap in the range of − 1 > *K*/*J* > 1/2^27^. The black, blue, and red circles indicate the bulk, edge, and corner states, respectively. The three corner states (the 16th to the 18th) have the same eigenfrequency *ω*_0_ because the corner states of a breathing kagome lattice are zero mode. The spatial distributions of the eigenstates are shown in Fig. 1c–f, respectively, where squared absolute values of the amplitudes of the eigenstates are integrated over the 1st to the 15th, the 16th to the 18th, the 19th to the 26th, and the 27th to the 30th. We observe large mode-amplitudes of resonators at the three corners (Fig. 1d), and at the three sides (Fig. 1e). Thus, the emergence of the corner and edge states is expected by using the above design parameters. For other eigenfrequencies, the bulk states are observed, where resonators around the center of the system have large amplitudes (Fig. 1c,f).

### Relation between admittance spectra and eigenstates

**Figure 2:**
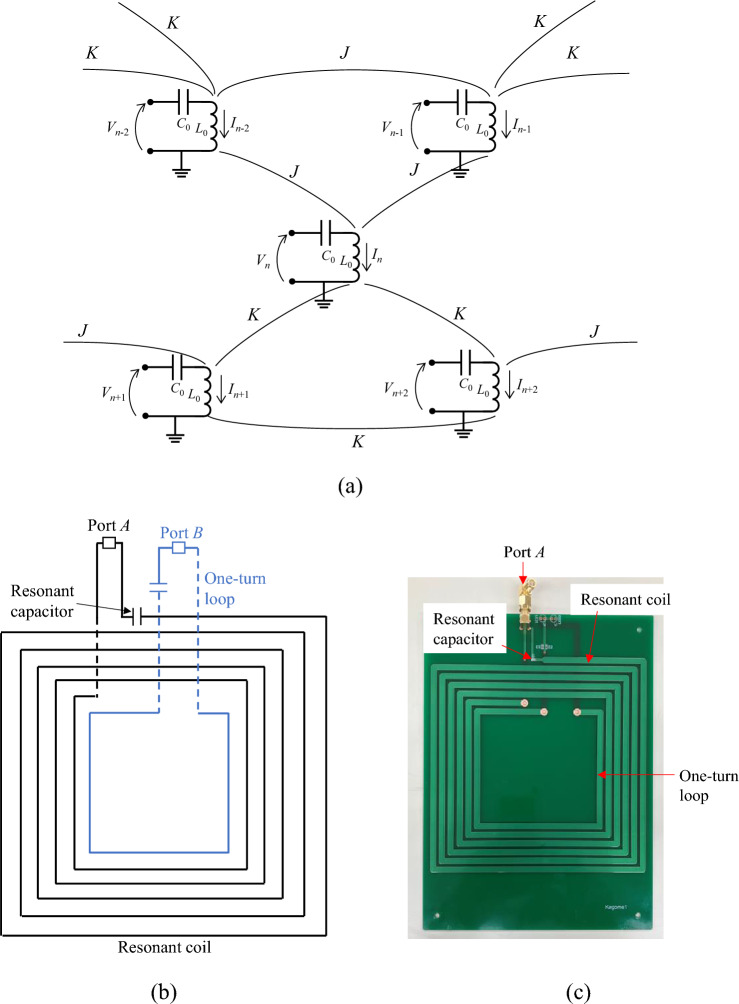
(**a**) Circuit diagram of magnetically coupled resonators. *L*_0_ and *C*_0_ are the inductance and capacitance of resonant coils. *V*_n_ and *I*_n_ are the voltage and the current of the *n*th coil. *K* and *J* are intra- and inter-cell couplings, respectively. (**b**) Schematic of the fabricated resonant coil. The solid and dashed lines indicate the copper film printed on the front and the back of the substrate. Black and blue lines correspond to the resonant coil and the one-turn loop, respectively. (**c**) Photograph of the fabricated resonant coil.

Here, we experimentally probe eigenstates through admittance measurements. Figure 2a shows the circuit diagram of our system. Our circuit consisting of magnetically coupled *LC* resonators is a dual of the capacitively coupled resonators^40^. The real part of the impedance of the capacitively coupled *LC* resonator circuit network corresponds to the density of states of the admittance matrix^40–42^. Thus, the real part of the admittance is used to probe eigenstates of the impedance matrix in this study. According to Kirchhoff’s law, voltage **V** and current **I** satisfy:

2a $$\mathbf{V}=j\omega {L}_{0}\left[H-\frac{{\omega }_{0}^{2}}{{\omega }^{2}}E\right]\mathbf{I},$$

2b $$H=\frac{1}{{L}_{0}}{\left(\begin{array}{cccccc}1& {\mu }_{K}& {\mu }_{K}& 0& 0& \cdots \\ {\mu }_{K}& 1& {\mu }_{K}& -{\mu }_{J}& 0& \cdots \\ {\mu }_{K}& {\mu }_{K}& 1& 0& 0& \cdots \\ 0& {\mu }_{J}& 0& 1& {\mu }_{K}& \cdots \\ 0& 0& 0& {\mu }_{K}& 1& \cdots \\ \vdots & \vdots & \vdots & \vdots & \vdots & \ddots \end{array}\right)}_{N\times N},$$

where **V** and **I** are *N*-vectors and ${\omega }_{0}=1/\sqrt{{L}_{0}{C}_{0}}$ (*L*_0_ and *C*_0_ are the inductance and the capacitance of the resonators). *E* is the identity matrix. ${\mu }_{K}$ and ${\mu }_{J}$ are the magnetic coupling coefficients for intra- and inter-cell couplings, where ${\mu }_{K}={M}_{K}/{L}_{0}$ and ${\mu }_{J}={M}_{J}/{L}_{0}$ with ${M}_{K}$ and ${M}_{J}$ being the mutual inductances for the intra- and inter-cell couplings. For the admittance measurement on the *n*th resonator, the other voltage nodes are short-circuited. The real part of the admittance *Y*_n_ at node *n* with other nodes being short-circuited is expressed as:

3 $${\text{Re}}\left[{Y}_{n}\left(\omega +j0\right)\right]=\frac{1}{2f{L}_{0}}{\sum }_{i}\delta \left({\lambda }_{i}^{H}-\frac{{\omega }_{0}^{2}}{{\omega }^{2}}\right){\left|{\psi }_{i,n}\right|}^{2},$$

where ${\lambda }_{i}^{H}$ and ${\psi }_{i,n}$ are the eigenvalue and the *n*th component of the *i*th eigenmode of *H*, respectively. *f* is the frequency 2π. The detailed derivation of the relation between the admittance and the density states are shown in Supplementary Information. Therefore, eigenfrequencies can be measured from the divergence of the real part of the admittance spectrum.

### Experimental setup

The schematic and photograph of a fabricated resonator are shown in Fig. 2b,c, respectively. The spiral-shaped copper winding is printed on a FR4 substrate as the resonant coil. A ceramic capacitor with a capacitance of 470 pF is loaded to obtain the *LC* resonance. The substrate has two ports; one port (labeled by *A*) is used for the admittance measurement, where port *A* is connected to the resonant coil. The other port (labeled by *B*) is used for the demonstration of wireless power transfer. Port *B* is connected to the one-turn loop that is implemented inside the resonant coil. All the resonant coils have a 150 mm outer radius, 4 mm linewidth of the winding pattern, 2 mm gap between neighboring winding patterns, and 5 turns of the winding. The copper film and the FR4 substrate have thicknesses of 35 μm and 1.6 mm, respectively. A resonance frequency of 3.17 MHz was measured (see Supplementary Information). In the admittance measurement for the *n*th resonator, ports *A* for all other resonators except for the *n*th resonator are short-circuited (see Supplementary Information) while all the ports *B* of the one-turn loops are open circuited. In the case of the transmittance measurement for wireless power transfer, port *B* for transmitting (receiving) resonant coil is connected to the corresponding port of a vector network analyzer (VNA) while the other ports *B* are open-circuited. Ports *A* for all of the resonators are short-circuited (see Supplementary Information).

### Topological phase

**Figure 3:**
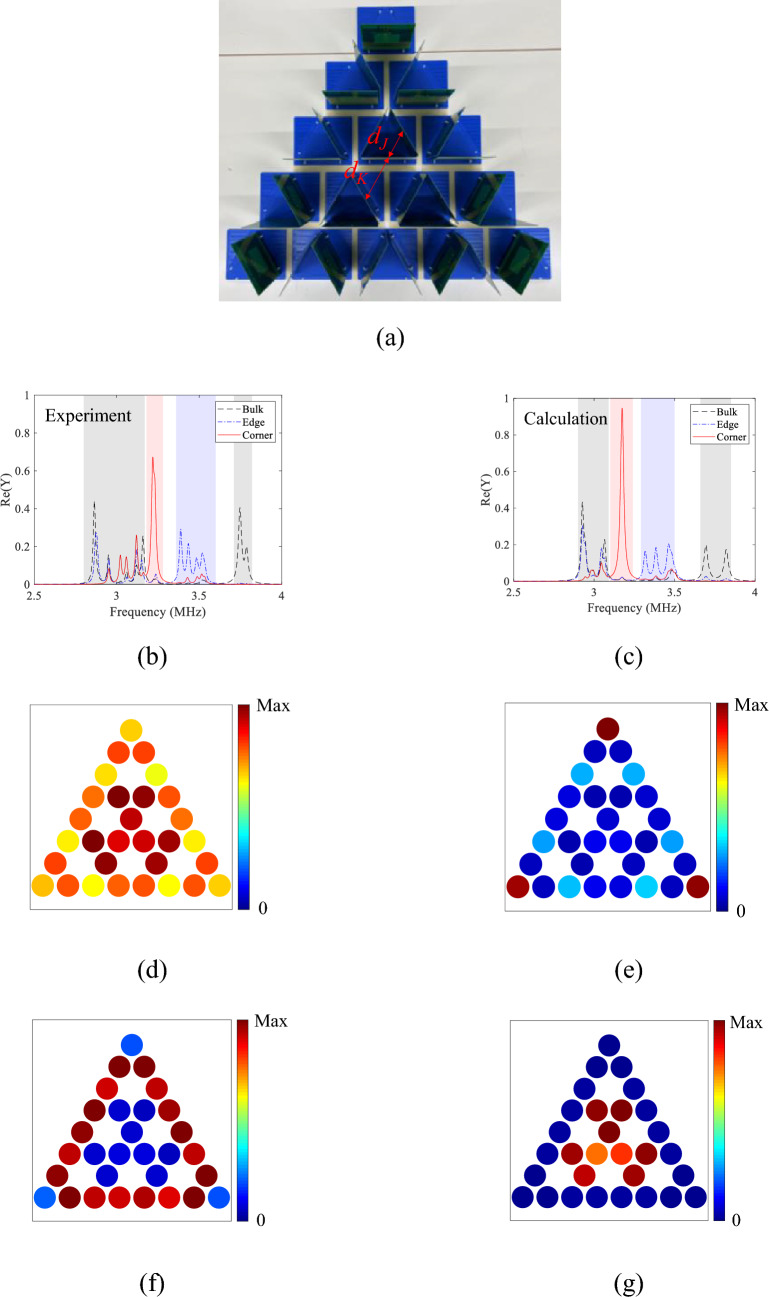
Breathing kagome lattice for the topological phase. (**a**) Photograph of the breathing kagome lattice consisting of the fabricated coils with distances ${d}_{K}$ = 13 and ${d}_{J}$ = 9 cm for intra- and inter-cell couplings. (**b**,**c**) Measured and calculated spectra of the real part of the admittances at *n* = 1 (corner), 10 (edge), and 13 (bulk), respectively. (**d**–**g**) Measured spatial distributions of the real part of the admittances integrated over (**d**) 2.80 to 3.17 MHz (bulk), (**e**) 3.18 to 3.28 MHz (corner), (**f**) 3.36 to 3.60 MHz (edge), and (**g)** 3.71 to 3.82 MHz (bulk), respectively.

The breathing kagome lattice consisting of fabricated resonators is shown in Fig. 3a. The center-to-center distance between coils for intra- and inter-cell couplings are ${d}_{K}$ = 13 and ${d}_{J}$ = 9 cm, respectively, so that the topological phase can emerge. The corresponding magnetic coupling coefficients are ${\mu }_{K}=-$ 0.053 and ${\mu }_{J}=$ − 0.123, respectively, as shown in Supplementary Information.

We measured the admittance spectra of all resonators. Figure 3b shows three representative admittance spectra measured at resonator *n* = 1 (corner), 10 (edge), and 13 (bulk). We observe the strong peak (red solid line) around 3.2 MHz for resonator *n* = 1. The admittance is integrated over the frequency range from 3.18 to 3.28 MHz (pink shaded region in Fig. 3b) for each of resonators and the spatial distribution is shown in Fig. 3e. Large admittances are observed at the three corners of the lattice (*n* = 1, 20, 30), validating the emergence of the edge states. The frequency of the peak is almost the same as *ω*_0_ since the corner mode is zero mode. Likewise, there are the peaks (blue dashed-dotted line) in the admittance spectrum from 3.3 to 3.6 MHz (blue shaded region in Fig. 3b) for resonator *n* = 10 which is positioned at the side of the lattice structure. As shown in Fig. 3f, the integrated admittances are localized on the three sides of the triangle lattice, indicating that the frequency band corresponds to the edge state. For resonator *n* = 13, peaks are distributed in the admittance spectra from 2.80 to 3.17 MHz, and from 3.71 to 3.82 MHz (gray shaded region). In the corresponding spatial distribution of the admittance, resonators around the center of the lattice have large amplitudes (Fig. 3d,g) for those frequency bands, indicating that those bands are the bulk state. The measured spectra agree well with the theoretical calculation using Eq. (S15) in Supplementary information, as shown in Fig. 3c. Parameters of *L*_0_ = 5.35 μH, *C*_0_ = 470 pF and* R*_0_ = 730 mΩ are used for the calculation, where *R*_0_ is the equivalent series resistance of the coil. The similar spatial distributions with the measured admittance are observed in the calculation based on CMT.

### Trivial phase

**Figure 4:**
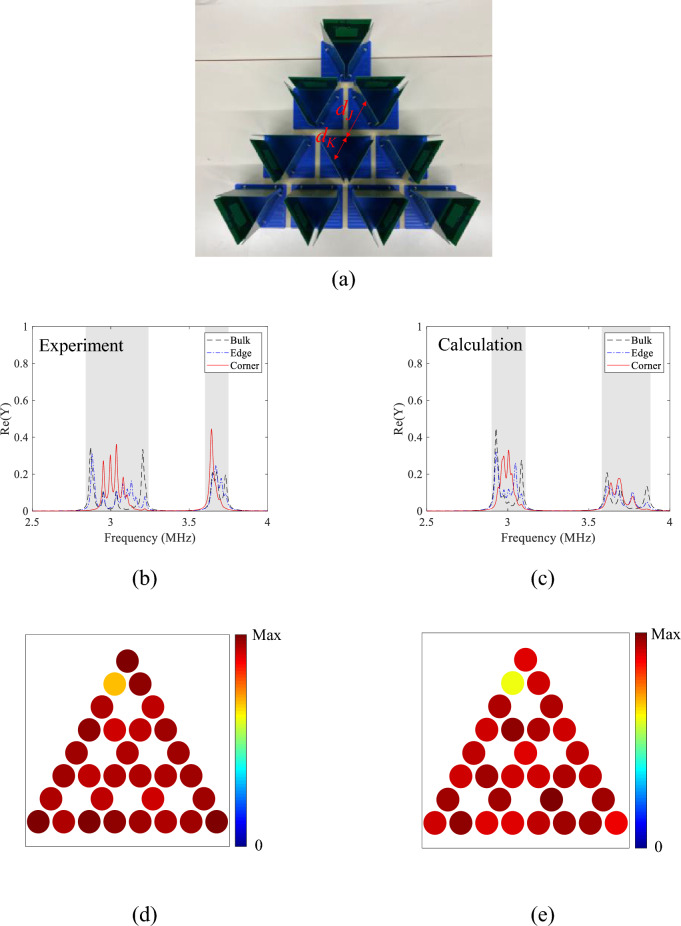
Breathing kagome lattice for the trivial phase, where distances between coils for intra- and inter-cell couplings are swapped from the system of Fig. 3 (${d}_{K}$ = 9 and ${d}_{J}$ = 13 cm). (**a**), Photograph of the breathing kagome lattice consisting of the fabricated coils. (**b**,**c**)**,** Measured and calculated spectra of the real part of the admittances at *n* = 1 (corner), 10 (edge), and 13 (bulk), respectively. The calculation is based on Eq. (3). (**d**,**e**)**,** Measured spatial distributions of the real part of the admittances integrated over (**d**) 2.84 to 3.24 MHz and (**e**) 3.60 to 3.75 MHz, respectively.

As a comparison, the trivial phase of the breathing kagome lattice is configured by changing the relative positions of the coils as shown in Fig. 4a. The distances ${d}_{K}$ and ${d}_{J}$ are swapped from the topological phase (${d}_{K}$ = 9 and ${d}_{J}$ = 13 cm). Figure 4b shows the representative admittance spectra measured at *n* = 1 (corner), 10 (edge), and 13 (bulk). For resonator *n* = 1, a peak is not observed around 3.2 MHz where the strong peak is observed in the topological phase. Similarly, no peaks are observed for resonator *n* = 10 in the frequency range of 3.36 to 3.60 MHz where peaks are observed in the topological phase. On the other hand, similarly to the topological phase, peaks are distributed in the admittance spectra from 2.80 to 3.17 MHz, and from 3.71 to 3.82 MHz (gray shaded region) for resonator *n* = 13. The measured spectra agree well with the theoretical calculation using Eq. (S15) in Supplementary information, as shown in Fig. 4c. The spatial distributions of the admittances are shown in Fig. 4d,e, where the admittance is integrated over 2.84 to 3.24 MHz for the lower frequency range, and 3.60 to 3.75 MHz for the higher frequency range, respectively. The admittances are distributed over the lattice in both frequency ranges, which is the same as the calculated eigenstates based on CMT shown in Fig. S1b and c. Thus, the corner and edge states are experimentally confirmed to be the features of the topological phase. Slightly smaller admittances for resonator *n* = 2 may be attributed to positional errors arising from a tilt of the substrate.

### Demonstration of wireless power transfer

**Figure 5:**
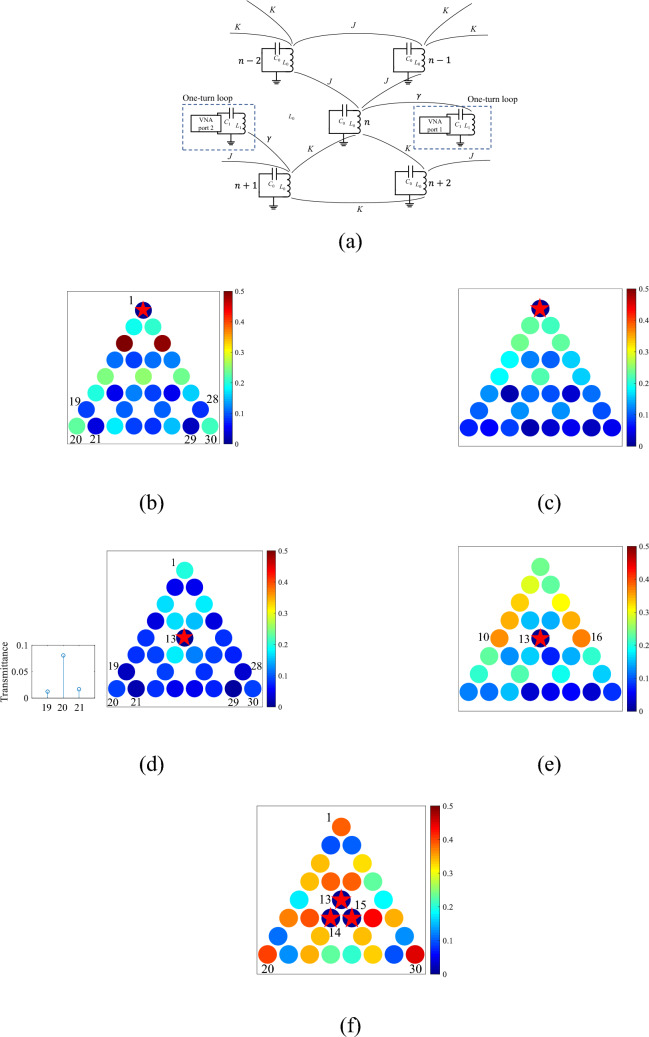
Demonstration of wireless power transfer. (**a**), Circuit diagram for the measurement. (**b**–**e**), Spatial distributions of the measured transmittances when resonator *n* = 1 is excited for (**b**) the topological phase and (**c**) the trivial phase, and resonator *n* = 13 is excited for (**d**) the topological phase and (**e**) the trivial phase, at the corner mode frequency (3.22 MHz) for (**b**) and (**d**) and the bulk mode frequency (2.95 MHz) for (**c**) and (**e**). The inset of (**d**) shows the transmittance for *n* = 19, 20 and 21. (**f**), Simultaneous excitation of three resonators *n* = 13, 14, and 15 (bulk three coils) at the corner mode frequency (3.22 MHz). The star markers indicate the excited coils.

Finally, we demonstrate wireless power transfer using the corner states. Figure 5a shows the circuit diagram of our wireless power transfer system. Each of two ports of a VNA is connected to port *B* of the one-turn loop, respectively, with each loop coupled to the resonant coil via magnetic coupling constant *g*. A capacitor with *C*_1_ = 7.8 nF is loaded in series with each of the one-turn loops, respectively, to make those loops resonant at the resonance frequency *ω*_0_^43^. The transmittance between the excitation and the receiver one-turn loops are measured by the VNA. The excitation one-turn loop is fixed at resonator *n* = 1, and the spatial distributions of the transmittance are measured by changing the location of the receiver one-turn loop. The spatial distributions in the topological and trivial phases are shown in Fig. 5b,c, respectively. The star marker indicates the position of the excited loop. In the trivial phase, the transmittance decreases as the receiver one-turn loop is away from the excited one-turn loop. In contrast, in the topological phase, the transmittances at resonators *n* = 20 and 30 from resonator *n* = 1 are larger than those at resonators *n* = 19, 21, 28, and 29, reflecting the feature of the corner mode.

Then, the bulk excitation, where the one-turn loop coupled to resonator *n* = 13 is connected to the VNA, is demonstrated as shown in Fig. 5d,e, respectively. In the trivial phase (Fig. 5d), the transmittances near the excited resonator *n* = 13 (i.e., *n* = 10 and 16) are large. On the other hand, in the topological phase, the transmittance to resonator *n* = 1 has the largest value. Though the transmittances to *n* = 20 and 30 are lower than that to *n* = 1, the transmittances are larger than the neighboring sites (*n* = 19, 21, 28, 29), where the feature of the corner states is observed (inset of the Fig. 5d). The lower transmittances for resonators *n* = 20 and 30 than for *n* = 1 may be due to the larger distance from the excited resonator.

## Discussion

The topological phases of a breathing kagome lattice in the electric *LC* circuit have been probed in impedance spectra^32,33^. In Refs.^32,33^, the grounded inductors are coupled via discrete capacitors. On the other hand, controlling coupling between the inductors is difficult by using the discrete capacitors. The advantage of our scheme using magnetic coupling is the reconfigurability.

Another class of corner states can appear above and below the edge band in a breathing kagome lattice with next-nearest-neighbor (NNN) couplings^28^. In our configuration, the NNN couplings are negligibly small. The coupling constant of NNN couplings in this study is 0.01*ω*_0_ as shown in Supplementary Information. Supplementary Figure 4c shows the calculated eigenfrequencies considering the NNN couplings. The type II corner states do not appear in the band gaps.

We adopted the coaxial arrangement^44^ of coils in 2-dimension rather than the planner arrangement^45^. Although the planer arrangement is another candidate for the breathing kagome lattice configuration, generally, the coupling between coils in the coaxial arrangement is stronger than the planer arrangement due to large flux linkage in the coaxial arrangement^38^. Thus, by using the coaxial arrangement, admittance peaks are more clearly distinguished, and a wide range of coupling constants is obtained. Additionally, long-distance magnetic fields can be improved by increasing the size of the coils (See Supplementary Information).

In the bulk excitation, the transmittance in the wireless power transfer to the nearest corner is stronger than the other two corners. The equivalent power transfer to the three corners can be realized by the excitation of the center bulk unit cell. Figure 5f shows the transmittance mapping with the excitation of the three bulk resonators. The transmittances are measured by using four ports of the VNA where one of the ports is coupled to a resonator and the other three ports are coupled to three resonators (*n* = 13, 14, 15) via one-turn loops. The transmittances for the three corners (*n* = 1, 20, 30) are almost the same as shown in Fig. 5f. We have verified that the spatial distribution of the transmittance in Fig. 5f is the superposition of the three cases where each resonator *n* = 13, 14, and 15 is excited.

Different ports were used for admittance measurement and wireless power transfer. To avoid decreasing Q factors of the transmitting and receiving coils^43^, we used one-turn loops for the wireless power transfer demonstration. On the other hand, the one-turn loops for wireless power transfer measurements were excluded in the admittance measurement so that the admittance of the resonators was measured directly to reflect Eqs. (2a) and (2b).

The proposed configuration is promising for an alternative scheme for selective wireless power transfer. Especially, by using the corner state, the power transfer to the intermediate coils can be suppressed. Thus, our wireless power transfer system using topological corner states can be applied to the countermeasure of the electrical energy theft^46^.

Our scheme enables the topological phase transition in a breathing kagome lattice by tuning the distances of resonators. Our reconfigurable platform is expected for investigating exotic topological phases of matter and will provide crucial understanding of topological phenomena. In addition, beyond basic studies, our scheme can trigger various applications of topological phenomena in electric circuits because the *LC* resonator is a fundamental circuit building block for such as oscillators^47^ and quantum electrodynamics devices^48^.

## Methods

We used multilayer ceramic capacitors (GCM series, Murata Manufacturing Co., Ltd.) as resonant capacitors for resonant coils, and one-turn loops. The impedance spectra were measured using an impedance analyzer (4294A, Agilent) with an impedance probe (42941A). The transmittances were measured using a four-port vector network analyzer (E5071C, Agilent).

## Supplementary Information

Supplementary Information.

## Data Availability

The data that support the present study are available from the corresponding author upon reasonable request.
