# Supplementary material for: Higher-order topological corner state in a reconfigurable breathing kagome lattice consisting of magnetically coupled LC resonators
Source: Sci Rep. 2023 May 23;13:8301. doi: 10.1038/s41598-023-35509-6 (PMC10205727; doi:10.1038/s41598-023-35509-6)
Supplement: Supplementary file 1 — Supplementary Information. [file 41598_2023_35509_MOESM1_ESM.pdf]

Supplementary Information for: Higher-Order Topological Corner State in a Reconfigurable Breathing Kagome Lattice Consisting of Magnetically Coupled *LC* Resonators

Kenichi Yatsugi[1*], Shrinathan Esakimuthu Pandarakone[1], and Hideo Iizuka[1]

[1]Toyota Central R&D Labs., Inc., Nagakute, Aichi 480-1192, Japan

Supplementary Note 1: Calculated eigenfrequencies and spatial distributions of eigenstates in the trivial phase

Supplementary Figure 1a shows the eigenfrequencies in the trivial phase calculated by coupled mode theory (CMT). The eigenfrequencies are split into two bands, where no eigenfrequencies are observed in the band gap. Supplementary Figure 1b and c show the spatial distributions of integrated eigenstates for the lower and upper bands, respectively. The eigenstates are distributed in the whole lattice in both bands.

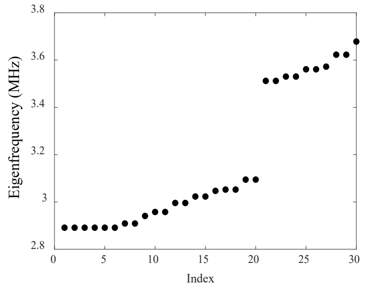

(a)

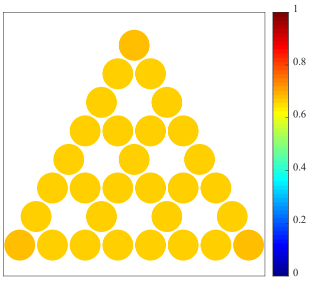

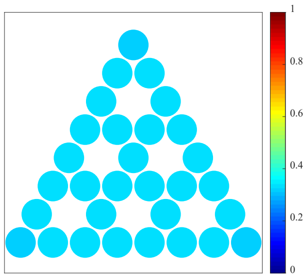

(b) (c)

Supplementary Figure 1. (**a**) Eigenfrequencies in the trivial phase calculated by using CMT. (**b**),(**c**). Spatial distributions of the eigenstates integrated over (**b**) the 1st to the 20th eigenfrequencies and (**c**) the 21st to the 30th eigenfrequencies.

Supplementary Note 2: Fabricated printed circuit board coil with port A being short-circuited

Supplementary Figure 2a and b show the schematic and fabricated printed circuit board coil with port *A* being short-circuited and removed. For efficient implementations, we have used the coils where the terminals of the spiral-shaped copper winding are short-circuited without the connector of port *A* (Fig. S2a,b), for the impedance measurement of the *n*th resonator (all resonators except for the *n*th resonator) and the wireless power transfer demonstration (all resonators).

Supplementary Figure 2c shows the impedance spectrum of the resonator shown in Supplementary Figure 2a and b. Supplementary Figure 2d shows the impedance spectrum of the resonator shown in Figure 2a and b. The resonant frequencies of both resonators are determined to be 3.17 MHz.

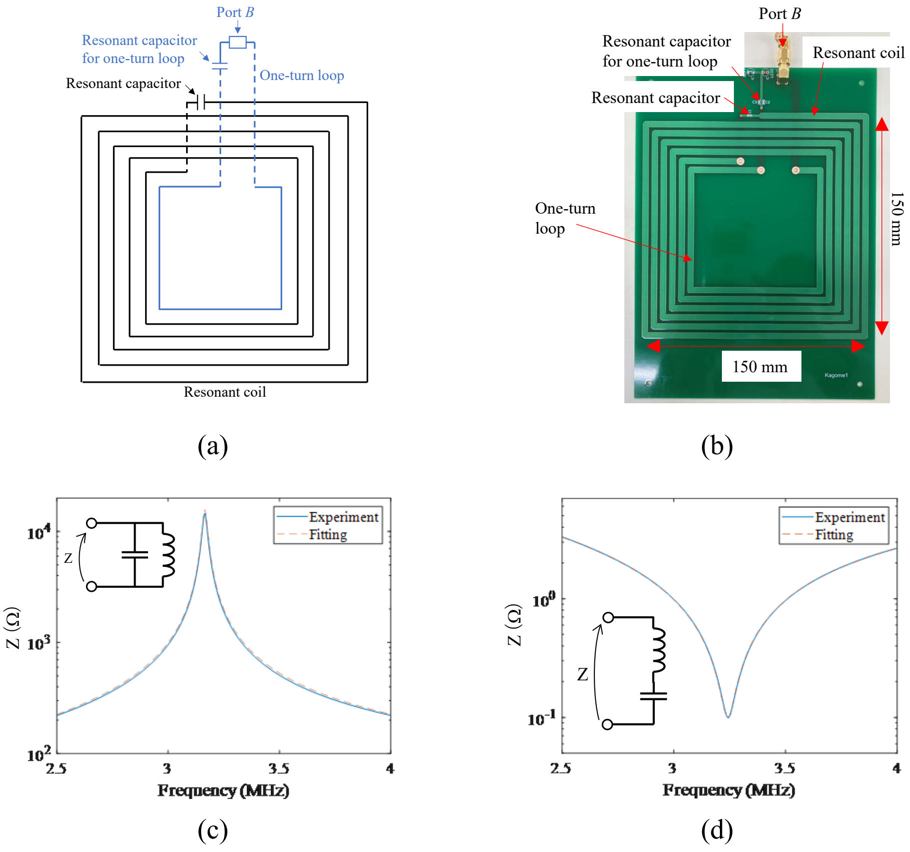

Supplementary Figure 2. (**a**), Schematic of the resonant coil where port *A* is short-circuited and removed. (**b**), Fabricated resonator where port *A* is short-circuited and removed. (**c**), Impedance spectrum of the resonator where port *A* is short-circuited and removed. (**d**), Impedance spectrum of the resonator with port *A*. The insets in (c) and (d) show the equivalent circuits.

Supplementary Note 3: Measurements of coupling constants

The coupling constants between resonators are deduced by fitting the impedance spectra using the mutual inductance as the fitting parameter. The equivalent circuit of the magnetically coupled coils is shown in Supplementary Figure 3**a**. The frequency dependence of the impedance is calculated as:

$$Z(\omega) = \frac{1}{j\omega C} + j\omega L - j\omega M + R + \frac{1}{(j\omega M)^{-1} + \left(\frac{1}{j\omega C} + j\omega L - j\omega M + R\right)^{-1}}. \quad \text{(S1)}$$

The configuration of the coils and the impedance spectrum $|Z(\omega)|$ for inter-cell coupling in the topological phase (intra-cell coupling in the trivial phase) are shown in Supplementary Figure 3b and c, respectively. Those for intra-cell coupling in the topological phase (inter-cell coupling in the trivial phase) are shown in Supplementary Figure 3d and e, respectively. The measured impedance spectra were well fitted by using C_0 = 470 pF and L_0 = 5.3 μF, and R_0 = 550 mΩ. The values of M_0 = -0.123L_0 and M_0 = -0.053L_0 are deduced by the fitting in Supplementary Figure 3c and e, respectively.

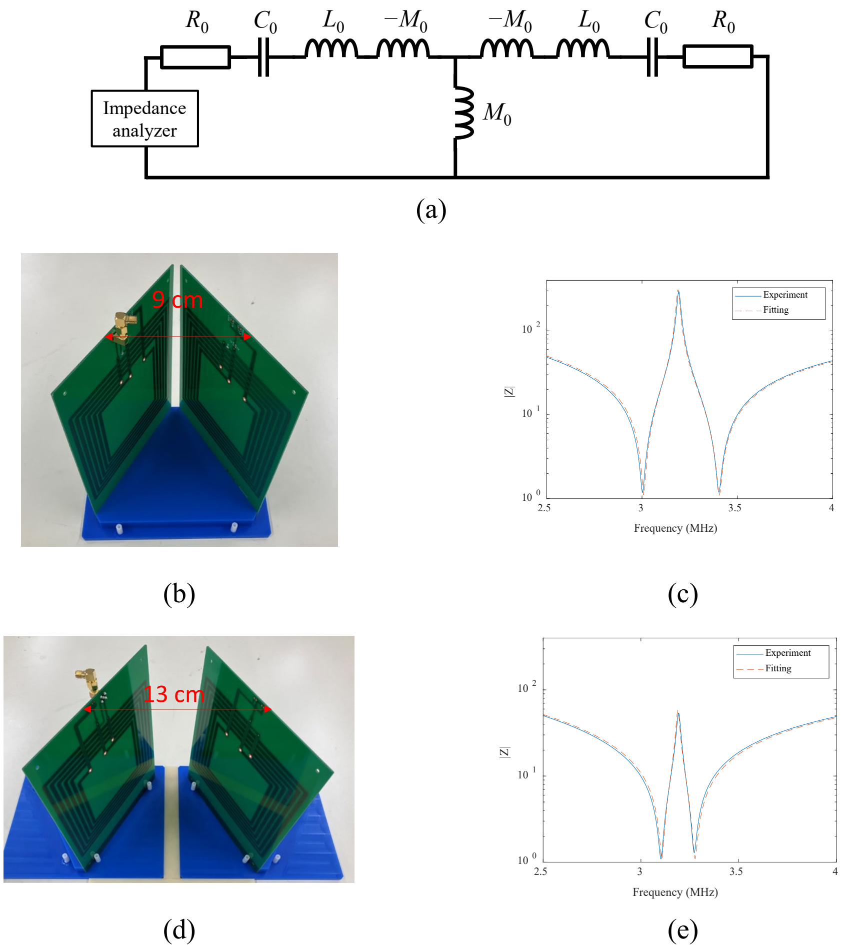

Supplementary Figure 3. Measurements of coupling constant. (**a**), Equivalent circuit for the coupling constant measurements. (**b**), Configuration for inter-cell coupling in the topological phase (intra-cell coupling in the trivial phase) and (**c**), the spectrum of the absolute value of the impedance. (**d**), Configuration for intra-cell coupling in the topological phase (inter-cell coupling in the trivial phase) and (**e**), the spectrum of the absolute value of the impedance.

Supplementary Note 4: Derivation of the relation of the admittance and the local density of state[2,3]

Here, we show the derivation of Eq. (4). In Eq. (2), $Z = j\omega L_0 \left[H - \frac{\omega_0^2}{\omega^2} E\right]$ is defined as the impedance matrix. We first introduce a matrix $Z(\varepsilon)$**,** by adding a small term εE to the impedance matrix Z, as

$$Z(\varepsilon) = Z + \varepsilon E, \tag{S2}$$

where E is the identity matrix with the same dimension as H. Then, we denote the inverse of $Z(\varepsilon)$ as

$$G(\varepsilon) = Z(\varepsilon)^{-1}. \tag{S3}$$

Multiplying $G(\omega)$ from the left to Eq. (2.a), we obtain $\mathbf{I}(\omega) = G(\omega)\mathbf{V}(\omega)$. Let U be the unitary

matrix which diagonalizes $Z(\omega)$ and Z,

$$U^{\dagger}ZU = \Lambda \text{ and } U^{\dagger}Z(\varepsilon)U = \Lambda(\varepsilon). \tag{S4}$$

Taking the inverse of the second equation, we have

$$U^{\dagger}G(\varepsilon)U = \Lambda(\varepsilon)^{-1}. \tag{S5}$$

It follows that we have

$$G(\varepsilon) = U\Lambda(\varepsilon)^{-1}U^{\dagger}. \tag{S6}$$

In the impedance measurements on the nth resonator, under the present experimental setup, the voltages on the resonators other than the nth resonator are 0, i.e., $\mathbf{V}$ has the form

$$\mathbf{V} = \begin{pmatrix} 0 \\ \vdots \\ V_n \\ \vdots \\ 0 \end{pmatrix}. \tag{S7}$$

Thus, from the relation of $\mathbf{I}(\omega) = G(\omega)\mathbf{V}(\omega)$, we have

$$I_n(\omega) = G_{nn}(\omega)V_n(\omega), \tag{S8}$$

where $I_n(\omega)$ is the nth component of $\mathbf{I}(\omega)$ and $G_{nn}(\omega)$ is the nth diagonal component of $G(\omega)$. Then, the admittance between node n and the ground, Y_n is given by

$$Y_n(\omega) = \frac{I_n(\omega)}{V_n(\omega)} = G_{nn}(\omega), \tag{S9}$$

where

$$\begin{aligned} G_{nn}(\omega) &= \lim_{\varepsilon\to 0}\sum_i \frac{|\psi_{i,n}|^2}{\lambda_i(\omega)+\varepsilon} \\ &= \lim_{\varepsilon\to 0}\sum_i \frac{|\psi_{i,n}|^2}{i\omega L_0\left(\lambda_i^H - \frac{\omega_0^2}{\omega^2}\right)+\varepsilon} \\ &= \lim_{\varepsilon\to 0}\sum_i \frac{\left[-j\omega L_0\left(\lambda_i^H - \frac{\omega_0^2}{\omega^2}\right)\lambda_i' + \varepsilon\right]|\psi_{i,n}|^2}{\left[\omega L_0\left(\lambda_i^H - \frac{\omega_0^2}{\omega^2}\right)\right]^2+\varepsilon^2}, \end{aligned} \tag{S10}$$

and $\lambda_i(\omega)$ is the eigenvalue of $Z(\varepsilon)$. From the relation of Eq. (S9), the real part of $Y_n(\omega)$ has the form of

$$\mathrm{Re}[Y_n(\omega)] = \lim_{\varepsilon\to 0}\sum_i \frac{\varepsilon|\psi_{i,n}|^2}{\left[\omega L_0\left(\lambda_i^H - \frac{\omega_0^2}{\omega^2}\right)\right]^2+\varepsilon^2}. \tag{S11}$$

By using the Dirac delta function of $\delta(x) = \frac{1}{\pi}\lim_{\varepsilon\to 0}\frac{\varepsilon}{x^2+\varepsilon^2}$, the real part of impedance $\mathrm{Re}[Y_n(\omega)]$ is written as

$$\mathrm{Re}[Y_n(\omega + j0)] = \pi \sum_i \delta\left[\omega L_0\left(\lambda_i^H - \frac{\omega_0^2}{\omega^2}\right)\right]\left|\psi_{i,n}\right|^2 = \frac{1}{2fL_0}\sum_i \delta\left(\lambda_i^H - \frac{\omega_0^2}{\omega^2}\right)\left|\psi_{i,n}\right|^2. \tag{S12}$$

Supplementary Note 5: Admittance calculation considering the resistance of the resonator

In the presence of a resistance for a resonance phenomenon, the impedance matrix Z is described as

$$Z = \left(j\omega L + \frac{1}{j\omega}W + R\right), \tag{S13}$$

where L, W and R are the $N \times N$ matrices of the inductance, the inverse of the capacitance, and the resistance, respectively. L is described as

$$L = \begin{pmatrix} L_0 & M_K & M_K & 0 & 0 & \cdots \\ M_K & L_0 & M_K & M_J & 0 & \cdots \\ M_K & M_K & L_0 & 0 & 0 & \cdots \\ 0 & M_J & 0 & L_0 & M_K & \cdots \\ 0 & 0 & 0 & M_K & L_0 & \cdots \\ \vdots & \vdots & \vdots & \vdots & \vdots & \ddots \end{pmatrix}_{N\times N}. \tag{S14}$$

The admittance between node n and the ground is calculated as[1]

$$Y_n = \sum_i \frac{\left|\psi_{i,n}\right|^2}{\lambda_i}, \tag{S15}$$

where $\psi_{i,n}$ is the nth component of the ith eigenvector of Z.

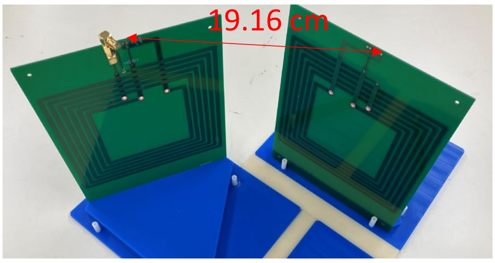

(a)

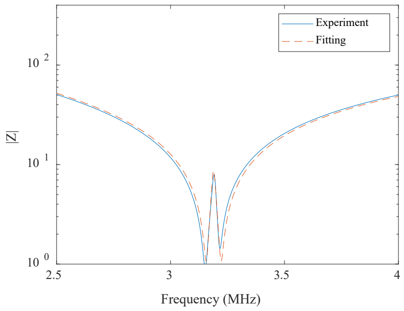

(b)

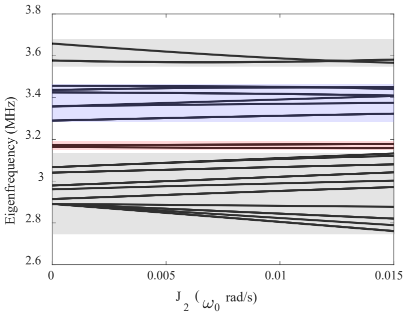

(c)

Supplementary Figure 4. Effect of next-nearest-neighbor (NNN) coupling. (a), Configuration for next-nearest-neighbor coupling. (**b**), Spectrum of the absolute value of the impedance. (**c**), Dependence of eigenfrequencies on NNN coupling constant (J_2). The bulk, edge, and corner states are highlighted in gray, blue, and red.

Supplementary Note 6: Effect of next-nearest-neighbor coupling

Supplementary Figure 4a shows the configuration of the coils for next-nearest-neighbor coupling J_2. The impedance spectrum is shown in Supplementary Figure 4b. The spectrum is well fitted with a mutual inductance of $0.02L_0$ ($J_2 = 0.01\omega_0$). The type II edge states are not observed above and below the band for the edge states. Supplementary Figure 4c shows the calculated dependency of eigenfrequencies on J_2 by using CMT.

Supplementary Note 7: Dependence of coupling constant on the coil size

Supplementary Figure 5a shows the simulation model created using Q3D Extractor (Ansys) for calculating coupling constant between two coils. Supplementary Figure 5b shows the distance dependencies of the coupling constant for different coil sizes. The experimental results agree well with the simulation results. The coupling constants increases as the coil size increases. Thus, the coupling constants for longer distance can be increased by increasing the size of the coil.

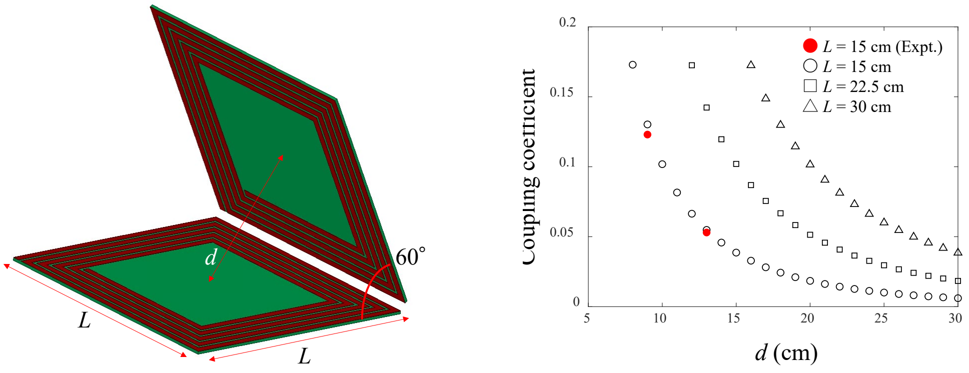

(a) (b)

Supplementary Figure 5. Simulation of coupling constant. (**a**), Simulation model. (**b**), Simulation results. The filled circles show the experimental results. Open circles, squares, and triangles show the simulation results for L = 15 cm, L = 22.5 cm and L = 30 cm.

Supplementary References

1. Imura, T. Wireless Power Transfer. Springer, (2020).

2. Wu FY. Theory of resistor networks: the two-point resistance. *Journal of Physics A: Mathematical and General* **37**, 6653-6673 (2004).

3. Wang Y, Price HM, Zhang B, Chong YD. Circuit implementation of a four-dimensional topological insulator. *Nat Commun* **11**, 2356 (2020).
